# Appointment

**Published:** 1862-08

**Authors:** 


Appointment.—C. B. Hutchins, M. D., Surgeon to the New York State Volunteers, with expectation of serving in the new regiment now being raised in Buffalo.

